# Faces in disguise. Masks, concealment, and deceit

**DOI:** 10.1007/s11245-022-09806-0

**Published:** 2022-06-17

**Authors:** Remo Gramigna

**Affiliations:** University of Turin, Torino, Italy

**Keywords:** Mask, Face, Dissimulation, Secrecy, Concealment, Disguise

## Abstract

The present study investigates and thematizes the interrelation between face masking, concealment, and deceit. It starts from the premise that the significance of disguise and deceit in the history of ideas should be reversed as these methods of the management of human appearance are not only regarded as coercive methods to manipulate and exert power over others but also as tactics skillfully used by the weak in order to outmaneuver those who are in a position of power. The study traces the matrix of simulation and dissimulation as forming the structure of deceit, it reviews some of the main theories of disguise within the field of semiotics, and it singles out two main dimensions of disguise, one geared upon dynamism and the other based on the static features of the face. This study suggests that classifications of masks elaborated in semiotic theory hitherto are useful but insufficient to encompass the full scope of such phenomenon. For this reason, the study provides a new typology of masks.

## The survival significance of disguise

*Mundus vult decipi* wrote Kierkegaard. The world wants to be deceived. Forms of disguise, concealment, and deceit occur in nature as well as in socio-cultural domains, revealing the ubiquity of these phenomena in our experience, affecting all compartments of life. Because deception is endemic to the human condition, evaluating what is true and what is false is something we all have to do. Indeed, the discernment of reliable sources and accurate information is a skill that has been essential for the evolutionary history of man and other species (Sommer 1992; Trivers 2011), and it will probably be a vital asset for the future existence of humanity. Both for human and non-human animals, assessing the reliability of signs is essential in coping with the environment. Moreover, signals and cues regarded as reliable often influence the structure, formations, and maintenance of people’s beliefs (Morris 1946, p. 121). As George Steiner pointed out, disguise has significance for survival:

Fiction was disguise: from those seeking out the same waterhole, the same sparse quarry, or meagre sexual chance. To misinform, to utter less than the truth, was to gain a vital edge of space or subsistence. Natural selection would favor the contriver… Loki, Odysseus are very late literary concentrations of the widely diffused motif of the liar, of the dissembler elusive as flame and water, who survives (Steiner 1975, p. 224).

Concealment and deceit are different phenomena, although they share something in common. Their mutual relationship is not so straightforward. Guy Durandin, in his study *Les fondements du mensonge* (1972, p. 20), pointed out a parallel between concealment and deceit. These strategies are used both for offensive (prevarication) and defensive purposes (survival). To hide oneself does not entail using language, nor does it imply the reference to a semantic notion of truth. As Harshorne and May pointed out (1928, p. 19), “the practice of deception is far older than language”. It is worth noting that, for Durandin, concealment is regarded as the model for all types of lies:

We shall first point out that a number of *animals* are able *to hide*, either to escape their enemies or to watch for their own prey and attack them by surprise. […] It may be objected that it is not a lie, because this behavior obviously does not imply words or scholarly reference to the notion of truth. But it plays a primary role in the struggle for life, whether in the form of defense or attack, and it is perhaps the source and model of any lie, since it consists in modifying the behavior of the adversary, by depriving him of the information he almost had: as soon as the predator no longer perceives his victim, he becomes incapable of pursuing it. And such is, in a quite general way, the process of lying: it is about transforming a situation to one’s advantage, by modifying the signs on which the judgment of the adversary is exercised.^1^

In this regard, the aspect of altering information is pivotal. The common feature between deception and concealment, thus, lies in that both phenomena tend to alter or distort the perception of a living organism via the foiling of the information gathered in order to gain a competitive advantage in the struggle for life. This point is important. As we shall see in what follows, this aspect has some considerable ramifications for the ways of managing one’s face. Indeed, disguise and masking in human relations are based on a similar principle, as they prevent face recognition.

Undoubtedly, there are different reasons and motifs behind employing strategies of concealment and deceit, but these operations occur on both fronts, defense, and attack. In the animal kingdom, this is commonplace. Numerous studies have dealt at length with “natural deception” (Hinton 1973) and have described the inner workings of camouflage and mimicry (Blechman nad Newman 2004; Maran 2017; Callois [1960] 1998). A similar logic applies to human relations. Indeed, camouflage and mimicry are also employed by people interacting in a real setting and they constitute a complex set of visual strategies used in various domains from art, to courtship, to war and politics (Behrens 2002; Casarin and Fornari 2010; Fabbri 2010; Bouvet 2001). As Karl Scheibe pointed out:

Logically, camouflage has the function of masking the presence of an individual or of objects, making them indistinguishable from their surroundings. The chameleon blends its coloration with that of its background to escape the alert eye of the predator. To a degree, human beings are capable of blending with their surroundings, becoming inconspicuous when they have reason to escape some danger or merely wish to avoid being conspicuous, as in the case of the Prince who became a Pauper (Scheibe 1979, p. 68).

A case in point is the management of people’s appearance. The human face plays a pivotal role in human relations for a host of different reasons, and therefore, it is not surprising to find that faces are pivotal to the management of appearance. This is epitomized by the use of face masks, although disguise is not limited exclusively to this aspect—hiding/altering facial features—but encompasses a host of different operations:

The physical implements of masking include actual face masks, cosmetics, costumes, practiced postures, gaits, airs, and attitudes. Also included are accents, intonations, and manners of expression. Hairstyle and facial hairstyle in men are also performative masks. The function of the mask is the management of appearances (Scheibe 1979, p. 67).

This is important because, as we will see later, disguise in human relations takes on different forms, and it capitalizes on what can be termed as an extended notion of physiognomy, which includes in the management of appearance, not only the face but a whole host of different implements. To be sure, this idea was already envisaged in the old treatises devoted to physiognomy, particularly by J. C. Lavater and G. Lichtenberg (Lavater and Lichtenberg 1991). They considered the physiognomy of the subject in all its ramifications, from face to costumes, to the way people walk, sit and act (Courtine, Haroche 1992, pp. 67–68; Klages 1949).

While humans co-opted the strategies of camouflage used by non-human animals, language increases the complexity of this issue. To lie by using speech is a modality of misrepresentation of reality that is species-specific, culturally determined, and very nuanced.^2^ Ogden and Richards (1946, p. 49), in their seminal work, *The Meaning of Meaning*, underscored the significance of the problem while discussing the treachery of language and the implications of misdirection. Many scholars focused on deception and lying from the perspective of linguistics and the philosophy of language.^3^ However, as pointed out before, the practice of deception and disguise goes beyond language and is prior to the use of speech acts.

In literature and mythology, the *topos* of disguise is legion. The archetype of the *trickster* is a case in point. Cunning, opportunistic dexterity, creative lie, and the ability to change skin and appearance according to the circumstances are the hallmarks of the *modus operandi* of the trickster (Radin 1954, 1972; Hyde 1998; Miceli 1984; Portelli 2004). Among the most illustrious ancestors of this archetype are Ulysses, whom Homer in the Odyssey describes as a man full of cunning, and Prometheus, who stole fire from the Gods of Olympus on behalf of mankind, and many other illustrations could be added to this list (Tagliapietra 2001; Bettetini 2001; Hesk 2000). Famous for his ability to deceive, he is multifaceted. *Polytropos* is the one who literally has many modes of being, modes of acting, and behaving.

The trickster is the holder of that form of flexible intelligence, able to adapt to the changing circumstances, which the Greeks called *mêtis*, a term that means “measure” (from *métron*) and also “prudence”, “cunning” or “worldly wisdom” and, metaphorically, the “art of plotting”. Detienne and Vernant ([1974]1991, p. 46) refer to *mêtis* as a particular form of intelligence, flexible and shrewd rationality, alternative or complementary to logical rationality and exact calculus. It is a practical intelligence characterized by its plasticity and obliquity. As Detienne and Vernant ([1974]1991, p. 21) pointed out:

*Mêtis* is itself a power of cunning and deceit. It operates through disguise. In order to dupe its victim, it assumes a form that masks, instead of revealing, its true being. In *mêtis*, appearance and reality no longer correspond to one another but stand in contrast, producing an effect of illusion, *apátē*, which beguiles the adversary into error and leaves him as bemused by his defeat as by the spells of a magician.

In the history and the philosophy of lying and deception, however, to mislead another, cheat or lead someone astray are conducts that for centuries have been deplored, demonized, and regarded as unethical or harmful to oneself and others.^4^ For this reason, however, it would be profitable to suspend a moralistic judgment upon this issue and free this terminology from its moral overlay (Scheibe 1980).

Undoubtedly, the structure of deceit is asymmetrical (Mecke 2007). Altering the relationship between the parties in human interaction (in this case, the deceiver and the dupe) concerning the access to information as well as the distribution of power is an important aspect of this phenomenon, and it plays a pivotal role in competitive or antagonistic settings. The privileged position of the deceiver (both in terms of information and power), as compared to the one to whom the deceit is intended, compromises the balance between the participants of the interaction in favor of the one who orchestrates the deceit. In this respect, deception can be seen as having an intrinsic connection with violence. Deception perpetuates powerlessness in others by coercing them into believing an altered depiction of reality. This is particularly illuminated in the seminal study of Bok (2003 [1978], p. 27), where deceit and violence are “the two forms of deliberate assault on human beings”. The privilege inherent in the deceit, as has been described above, affects the exercise of power in an identical measure in which the access to knowledge confers supremacy. Therefore, for Bok, there is a biunivocal correspondence between deceit and power. Deception manifests an insidious influence through manipulating information to the extent that it coerces the choice-making process of the dupe.

However, there is more to it, and Bok’s view is not an unbiased estimate of how deception operates. Indeed, Bok seems not to take fully into account the historical evidence that deceit and cunning have been used as resources by the weak to outmaneuver those in a position of power and, thus, not only as a way to exert power over people. Indeed, the history of folklore is riddled with illustrations of the opposite tendency, namely, the victory of the weak over the strong by means of acumen, practical intelligence, and ultimately cheating. As Lotman (2009, p. 39) pointed out, “the wise man [sic] conquers by means of his resourcefulness, astuteness, craftiness and wiliness […] The wise man [sic] is he who accomplishes unexpected, unpredictable actions in the face of the enemy. Intelligence is actualised as astuteness”.

The crux of the question is so well laid out by Barton Whaley, who, by drawing on the sixteenth-century Florentine intellectual, Niccolò Machiavelli and his sharp distinction between ‘force’ and ‘fraud’, envisaged cheating in all its variations as a strategy used by the weak as a way of self-defense against the strong: “the question is one of brute force versus dissembling, cunning, guile, fraud—in short, force versus deception” (Barton Bowier 1982, p. 4).

The author provides numerous historical examples to substantiate this claim. He refers to the case of the black slaves in America in the pre-Civil war, who developed a way of silent resistance to gain leverage against their masters: “the slaves’ strategy, called “masking”, involved projecting a false personality to deceive the masters. Most enslaved people deliberately feigned passivity, laziness, stupidity—the Sambo image. This largely successful strategy constitutes a type of passive resistance” (Barton Bowier 1982, p. 5). Similarly, the Chinese political defectors who traveled to Japan in the ninth century and were hunted by the Japanese Shinto religion developed a set of skills for survival termed *niniutsu* (ibid.). This term literally means “hiding” or “the art of invisibility”, and the holders of this secret clan were termed the *ninja* (“hider”):

Masters of concealment, ninjas *never* appeared outside their own clans without disguise—as priests, craftsmen, itinerant tradesmen, enemy soldiers, anything but what they were. Moreover, disguise extended beyond the mere donning of costume to careful mastery of the customs, gestures, postures, and jargon associated with the role. The ninja also mastered camouflage, blending with the night in black coveralls with all-black equipment or blending with the snow in white uniforms with white equipment (Barton Bowier 1982, p. 6).

The strategies of cunning and concealment, thus, not only are used as a means of attack or as a means to control and exert power and violence over people but is also part of the “strategic armamentarium” (Scheibe 1979, p. 53) of the prey who seeks to render itself invisible to the hunter, as well as the weak person who seeks to outsmart the strong or simply seeks to be undetected and unrecognized. In other words, disguise, masking, and other forms of manipulation of human appearance can also be couched in terms of resourceful tactics used to be undetected, invisible, unnoticed, and not only as a way to deceive. Not surprisingly, protection via concealment is one of the major functions of masking (Scheibe 1979, p. 67).

Summing up, disguise can be thought of as a tactic used by the weak as a resource to outsmart the strong. In what follows, I will discuss how this occurs. As a way of putting things into perspective, it should also be pointed out that disguise as *modus operandi* resurfaces today under various guises. In this respect, it suffices to mention that hiding and disguise have been recently used to prevent detection of the face by artificial intelligence technology. People used masks and costumes to avoid the possibility of recognition, as masks obfuscate the informative indices coming from the face. Indeed, recent literature on the subject has suggested the relevance of face disguise in preventing automatic face recognition (Noyes and Jenkins 2019).

## To hide or not to hide: the matrix of showing and concealment

Communication scholars, as well as psychologists, have focused on the concept of information as the benchmark for the study of deception (Knapp and Comadena 1979; Bavelas et al. 1990; Scheibe 1979). As said before, disguise, masking, and camouflage manipulate information by altering and foiling the information gathered. Information management is a complex phenomenon ruled by a logic of concealment and revelation.

Indeed, one common theme runs through the study of this subject, namely, the distinction between ‘simulation’ and ‘dissimulation’, which are terms often used in the scientific literature to distinguish between two separate and yet complementary modalities of altering the perception of reality. In a short essay entitled “Of Simulation and Dissimulation” (1838), the English philosopher Francis Bacon drew attention to the matrix of showing and concealment. Bacon cataloged simulation and dissimulation among the strategies of hiding oneself and laid out a three-fold typology:

There be three degrees of this hiding and veiling of a man’s self. The first, closeness, reservation, and secrecy; when a man leaveth himself without observation, or without hold to be taken, what he is. The second, dissimulation, in the negative; when a man lets fall signs and arguments, that he is not that he is. And the third, simulation in the affirmative; when a man industriously and expressly feigns and pretends to be that he is not (Bacon 1838: 387).

Secrecy, dissimulation, and simulation are, thus, for Bacon, the three degrees of hiding and veiling oneself, and they should be kept distinct on an analytic level, although there are relations between them. Bacon’s threefold gradation is a typology in which each degree of hiding is ranked on the basis of the degree of secrecy it possesses, as well as on the type of culpability of the habit. Hence, while the first species of hiding is the most excusable, the third degree is the most culpable and the less politic. Bacon’s classification, thus, is predicated upon a descending order (Table 1):

**Table 1:** *Bacon’s degrees of hiding*

1. Secrecy	A man makes himself invisible	Neutral position
2. Dissimulation	A man gives evidence that is something other than himself	“Negative” position
3. Simulation	A man feigns and pretends to be that is not	“Positive/affirmative” position

Whilst secrecy is the making of oneself invisible and, thus, it is a more neutral stance—a sort of zero degree, as it were, or hiding oneself—dissimulation and simulation oscillate between two opposite poles: the negative and the positive (Table 2).

**Table 2:** *The poles of positive and negative as applied to simulation/dissimulation*

Dissimulation	Secrecy	Simulation
-	0	+

Whilst dissimulation is aimed at not letting others know by means of signs or arguments that he or she is something other than himself/herself and entails a “negative” position, simulation is to feign and pretend to be what one is not and entails a “positive” position by affirmation.

As pointed out before, the duplet simulation/dissimulation is common in the scientific literature, despite the variety of the terminology used by each scholar. A useful list (Table 3) of the conceptual and terminological differences in describing how information is altered can be found in Vincent Marrelli (2004), which I took the liberty to arrange in a more systematic way, and I have included some additional sources:

**Table 3:** *Adapted from Vincent Marrelli* with my own additions (2004, p. 180)

Chisholm and Feehan (1977)	Commission	Omission
Grotious (1641)	*Suggestio falsi*	*Suppressio veri*
Nyberg (1993)	Showing	Hiding
Barton Bowyer (1982)	Revealing	Concealing
Castelfranchi and Poggi (1998)	Creating the false	Hiding the real
Anderson (1985)	Distortion of information (deceit)	Suppression of information (secrecy)
Accetto (1641)	Simulation	Dissimulation
Duprat (1903)	Positive suggestion	Negative suggestion
Bacon (1625)	Simulation	Dissimulation

Numerous scholars have considered deception as predicated upon the duplets hiding/showing, concealment/revelation, simulation/dissimulation, opacity/transparency and have envisaged these poles as two sides of the phenomenon: one is covert and hides the real (dissimulation), the other is overt and shows the false. Despite these being two facets of the problem that are set aside for analytical purposes, they operate in tandem so that, in practice, they are interrelated. Both dissimulation and simulation entail the use of different techniques cataloged, with incongruent results, by many scholars.

Whaley, for instance, singles out three ways to dissimulate and three ways to simulate, one representing the counterpart of the other. Hiding the real can be done “by making it invisible” (*masking*), “by disguising” (*repackaging*), and “by confusing” (*dazzling*). Showing the false can be done “by having one thing imitate another” (*mimicking*), “by displaying another reality” (*inventing*), “by diverting attention” (*decoying*) (Whaley 1982, p. 185).

## Semiotic approaches to disguise, concealment, and deceit

In this section, I will limit my attention to framing disguise from the vantage point of semiotics. As Michel de Montaigne famously wrote in his *Essays*, the reverse of truth has a thousand shapes.^5^ The deception construct alone has several unavoidable overlaps with other forms of misrepresentation and distortion of reality that, despite having some features in common, must nonetheless be distinguished. With this understanding, the concept of deception has been widely extended to a host of complex phenomena—lying, simulation, fabrications (Goffman 1974), feigning, pretense, mimicry, hypocrisy, disguise, error, camouflage, masking, non-serious talk, to mention but a few. This overlapping, however, is often a source of confusion. Roget’s *Thesaurus* lists approximately eight hundred words similar to “deception” (Harshorne and May 1928, p. 19), and Vincent Marelli (2004, pp. 400–406) shows by lexical investigation a very robust corpus in English for the semantic field lying/deception.

Deception takes many forms. Lying can be considered one of them. Lying can be done by means of speech (Weinrich 2005), pictures and images (Nöth 1997; Roskill, Carrier 1983), gestures, and reticence (Courtine, Haroche 1992, pp. 138–155; Colish 1978; Mazzeo 1962; Volli 2020), and for some, by nonverbal language (Eco 1997). In principle, semiotics should have a privileged position in the study of phenomena such as deceiving, masking, and all the paraphernalia used to alter the perception of reality because lying would be altogether inconceivable without the use of signs (Eco 1975; Pelc 1992). As both J. P. Sartre and F. Nietzsche note, the concept of deceit is quintessential to the notions of sign and representation (Castelfranchi and Poggi 1998, p. 19). Likewise, Hanna Arendt was very sharp in pinpointing an element of creativity inherent within any form of fabrication. Indeed, the lie is interlocked with counterfactual imagination, the capability of imagining it possible in the world. As she pointed out: “The deliberate denial of factual truth – the ability to lie – and the capacity to change facts – the ability to act are interconnected; they owe their existence to the same source: imagination” (Arendt 1972, p.5).

A similar idea is nested in U. Eco’s depiction of semiotics as it was treated *ex professo* in his early treatise; “semiotics is the discipline studying everything which can be used in order to lie. If something cannot be used to tell a lie, conversely it cannot be used, to tell the truth: it cannot be used to tell at all” (Eco 1976, p. 7). This definition has often been repeated, and therefore, I feel exempted from going into too much detail. This said, one would expect that the natural development of semiotics would include the study of the semiosis of deception and that, broadly speaking, phenomena like erroneous inferences, lies, deceits, simulations, misperceptions, masking and all devices of misrepresentation would appear to be the proper domain of semiotics. In fact, quite the contrary is true. Regrettably, in comparison to philosophers, theologians, psychologists, linguists, journalists, and political scientists, semioticians have been concerned with this subject sparingly and did not quite keep up with advancements made in other fields. This fact is astounding, to put it mildly.

As Danesi (2017, p. 20) pointed out, “it is somewhat surprising to find that virtually no one has approached sign analysis from Eco’s perspective, even though it goes way back to 1976”. This claim is backed up by the paucity of semiotic research conducted on the subject, with the exception of a few studies Anderson 1986; Danesi 2014; 2020; Eco 1997; Fiordo 1990; Jervis 1970; Maddox 1984; Maran 2017; Nöth 1997; Nuessel 2013; Pelc 1992; Sebeok 1975).^6^ Undoubtedly, there is a need to bridge such a gap.^7^

### Semiotic typologies

Within semiotics, the most well-known signpost for the study of this subject is the so-called ‘square of veridiction’, also referred to as the ‘veridictory square’. Such a heuristic device, theorized by Algirdas J. Greimas and Joseph Courtés, “can be used to examine the dynamics of veridiction (truth and falseness) in a semiotic act, particularly a text” (Hebert 2006, p. 29), and it is based on the opposition between being/seeming articulated onto a semiotic square, which renders the following four-fold taxonomy:

- True or truth (being + seeming);
- Illusion or lie (non being + seeming);
- False or falseness (not being + not-seeming);
- Secret or dissimulation (being + not-seeming).

Greimas and Courtés pointed out that the categories assumed in a square are not to be considered ontological entities (Greimas 1979, p. 380). In other words, the authors refuse a claim of referentialism because they conceive of truth or falseness in terms of effects produced within the production of the discourses. However, Greimas and Courtés’s take is not without shortcomings. Indeed, the square of veridiction does not seem to take fully into consideration the distinction between errors or mistakes and intentional lies. Defining the ‘lie’ or the ‘illusion’ as the conjunction of seeming and not being, as the authors have proposed, blurs the distinction between unwilling mistakes and intentional untruthfulness, which is one of the lynchpins in the literature on lying/deception (Derrida 2002). Recent reformulations and important amendments to the square of veridiction as applied to the themes of secrecy and manipulation can be found in Volli (2020), Bertrand (1987), and Parret (1978). An application of Greimas to the issues of masks can be found in Marino (2021, p. 326).

A different approach geared towards pragmatics and not escaping engagements with ontology can be found in Eco. Whilst from its inception, semiotics was equated to the study of anything that can be used in order to lie, it is worth noting that elsewhere Eco recanted this stance (Eco 1997; 2017). Indeed, on several occasions, he pointed out that semiotics should not be conceived as a theory of lying but rather as a theory of how it is possible to say *what is not the case* (Eco 1997, p. 37). Indeed, there are many modalities to say what is not the case, and the lie is only one of them. In this connection, it is useful to recall that, from the vantage point of a general semiotic theory, Eco, in a rarely cited article, deals with masking and disguise in the context of a wider typology of cases in which it is possible to say (or better signify) *what is not the case*. There are several ways to say what is not the case. Eco (1997, p. 36–39) identifies three large categories that explain this phenomenon:

— To be mistaken;

— To deceive;

— To pretend.

To mistake or to be wrong about something encompasses two different phenomena: (1) misinterpreting, which has to do with the content, and (2) confusing one thing with another, which instead involves the level of expression. A classic example of an error of perception is that of the stick that appears broken when immersed in the water. The second category, to deceive, includes, in turn, two types:

— Lying;

— Falsifying.

For Eco, lying is a purely linguistic phenomenon and has to do with the referent. Eco’s typology can be seen in the table below.

**Table 4:** *U. Eco’s typology of what is not the case. * (adapted from Eco 1997, p. 34)

To be mistaken		To deceive			Non-deceptively intended pretense
*Misinterpreting (content)*	*Confusing one thing with another (expression)*	*Lying*	*Falsifying*	*Deceptively pretending*	*Theatrical masking*, *fictional narrative, modeling simulations*
		*Simulation (a behavioral form of lying)*		*Masks as pretending to pass off as another*	

It should be noted that Eco included in the category of deception the ‘simulation’, which he regarded as a nonverbal form of lying. He poses the following question: “What is the difference between the mask of Diabolik (which allows the bandit to simulate being another) and Pantaleone’s mask (which the actor wears out of fun and without the willingness to deceive us, but only to ‘pretend’?”(Eco 1997, p. 135).

From the aforesaid, two distinct uses and functions of masking can be extrapolated. The mask of Diabolik is a form of deceit that falls in the category of simulation as it allows the bandit to pass off as someone else, counterfeiting his own identity. In fact, simulation is conceived as a nonverbal form of deception that takes place through behavior and disguise. The mask Diabolik is wearing not only has a semantic value but also possesses the pragmatic effects of the sign on the sign-receiver and, therefore, has got pragmatic value (Eco 1997, p. 137).

The case of the theatrical mask is, however, a different matter. The mask of the actor is not used to deceive, but only to pretend to be a character on the stage.^8^ According to Eco, what is fictitious is based on a convention; pretending is not a real form of deception, but it is a form of para-deception. Thus, pretending does not intend to deceive and therefore does not have the pragmatic dimension of deceit. Pretending includes various phenomena, from theatrical masks to fictional narratives, from counterfactuals to modeling simulations and protensive simulations (Eco 1997, p. 137). Although Eco’s distinction of types of masking is relevant, it does not exhaust the alternatives that a typology of disguise should be able to encapsulate. I will come back to this point in the final section of this paper. Now, a brief discussion of what falsification is, in which I shall complement the outlook of Eco’s theory on the subject.

### Semiotic theory of falsification and forgery

Umberto Eco gave us the first formulation of a fully-fledged semiotic theory of falsification (Eco 1988, 1990, pp. 162–192). Eco set out a very sophisticated typology of fakes, of which I will lay out the main categories that can be useful for the subject treated in this paper. The first type of Eco’s semiotics of falsification is “doubles”. Doubles concern the replicability of an object, and they do not intend to deceive. A double is a physical occurrence that has got all the properties of another physical occurrence (Eco 1988, p. 69; Eco 1990, p. 165) Examples of doubles are two eggs or two sheets of white paper which can be used for the same purpose.

The second head in Eco’s typology is “pseudo-doubles”. There is a pseudo-double when one single occurrence takes on a particular value for one of the following reasons. Because of its origin, as in the case of the first car Model T produced by Ford, or because the object was used in a particular context, as in the example of the Holy Grail used by Jesus Christ (Eco 1988, p. 70). Now we have reached the third category of falsification and the most important point for the purpose of this paper: “false identification” (Eco 1988, p. 71; Eco 1990, p. 168). False identification may occur when there are two different objects – object A and object B – produced by two different authors in two different historical contexts. These two objects can be identified as being identical or indiscernible either by an individual or by a group of individuals who decide that the two objects are identical. This leads to a host of different problems.

First of all, the issue of intentionality. There are two different levels of intentionality at stake. Firstly, the intention of the people who produced object B. Secondly, the intention of the people who identified object B and object A as identical. If we consider the people who produced object B, we can distinguish a bona fide and mala fide intention. Author B might have produced object B maliciously to pass off something as fake for real, but he might also have acted in good faith. More complex is the situation of the intention of those who decide that object A and object B are identical. Considering the subject’s knowledge and beliefs adds complexity to the problem. According to the type of belief the individual or the group has, Eco singles out four different cases.

The first case is *confusion between identity and interchangeability*. Someone knows very well that object A cannot be identified with object B, which was produced afterward and by imitation, but believes that the two objects are interchangeable in value and function and uses and presents one as identical to the other. The second case is one of *malicious false identification* (Eco 1988, p.71). Someone knows that object B is just an imitation of object A and cannot be identified with it, nor does he believe that the two objects are interchangeable. But, in bad faith, he pretends (and says) that oB is identical to oA. There can also be false identification because of the *inexperience of the individual*. He ignores that the two objects are not identical. The fourth type is the *presumption of interchangeability*. Someone knows very well that oA and oB are physically different but decides that, under a certain description and for the purposes of a certain practice, one is as good as the other and is presented not as identical but as completely interchangeable. Eco’s theory of fakes and forgeries is much more detailed than it is represented here and it would deserve an *ex professo* discussion and critical examination.

## Physiognomic underpinnings of counterfeiting and falsifying the face

There is a turning point in the history of physiognomy that bears significance to the subject at hand. This rupture occurs in the debate around the human faces between two outstanding scholars: Johan Caspar Lavater and Georg Lichtenberg. There is a key distinction that can be drawn from this debate, namely, the difference between ‘physiognomy’ and ‘pathognomy’. While physiognomy is concerned with the study of fixed or static traits of the human face, pathognomy tackles the study of the traits of dynamic or mobile facial features and their expressions, emotions and affect. As Gurisatti (1991) pointed out, the pinnacle of this development was underscored in the XVII century debate between these two eminent scholars.

This point is worth pondering. The debate between Lavater and Lichtenberg not only marks a turning point in the history of physiognomy, but it also has some important ramifications for the subject of this paper for it shows two aspects of the study of the face–static versus dynamic–as well as two dimensions for grappling with the concept of disguise and understanding how it operates.

The breakthrough of Lichtenberg is quite a revolution. Probably for the first time, the discussion around the human face takes on a dynamic dimension, which entails the possibility of change and transformation, not only as external and environmental factors but also as intentionally couched by the individual in order to achieve certain effects. Hence, here there can be traced a link between the dynamic aspect of the face on the one hand and the capability of deceit, cunning, and disguise on the other, through the mimic resources of the skilled individual, who is able to control his or her physiognomic expression.^9^ For Lavater, physiognomy is thought of as the ability to recognize, from the outer appearance of a man, his inner aspects. He conceives physiognomy as the investigation of the fixed forms of the face and the body. For Lichtenberg, one should reject the study of the fixed forms of the face and replace it with the study of mobile facial expressions – known as “pathognomy” of “semiotics of the affects” – which reveals what man expresses or simulates to express in a given moment and in a specific context (Lichtenberg 1991, p. 108).

Between 1770 and 1775, Lichtenberg visited London, and during these years, he had the chance to study, very closely, the great actor David Garrick whose chameleon-like ability to change face through the art of dissimulation he greatly admired. *The Letters from England*, written by Lichtenberg, in fact, revolves around the actor, which is the quintessential element of transformation, mutability, and dynamicity of the face. The actor represents the chameleon-like man, as Gurisatti (2006, p. 98) calls it. In Lichtenberg, thus, there can be found semiotics of passions, feelings, and emotions. This represents the study of the natural signs of movements of the soul, in other words, the language of involuntary gestures and of mimic expressions. This approach is also plain in Charles Le Brun, in the field of pictorial representation, who sought to pin down a repertory of the expressions of facial emotions (Courtine, Haroche 1992, p. 64–68; Damisch 1992).

There is an important corollary that stems from the distinction between the study of static traits and the study of the dynamic traits of the human face. The lynchpin that should be drawn from Lichtenberg’s view, is that not only is the face the involuntary medium of an expression, but the face also becomes the voluntary means of the representation of the subject. In other words, the face is the tool used by the subject in order to dissimulate his thoughts, intentions, states of mind, and character (Gurisatti 2006, p. 107). As Lichtenberg writes: “The mobile features of the face show and enumerate not only the involuntary, pathognomonic movements but also the voluntary movements of simulation” (Lichtenberg 1991, p. 130). How to draw a line between intentional and more spontaneous expressions remains, however, a hard nut to crack.

Here we come full circle. For Lichtenberg, the face is regulated by the “I” – the individual – who can not only manage and control his own emotions and the respective mimic expressions – thus hiding something that exists inside and should not be revealed outside – but also producing a facial expression that is altered and artificial, thus, simulating that which does not exist inside and must show to the outside. Thus, with Lichtenberg is plain that the face is used as a device for dissimulation, as a mimicking and chameleon-like tool, which is epitomized by the skill and the talent of the actor and the man of the world (Gurisatti 2006, p. 210). Lichtenberg really hits the mark because he accounts for the ability to use the signs of expression in a strategic way and to capitalize on them. In this respect, falsifying and counterfeiting the face means the ability to control the facial mimic in order to project a false impression.

We can, thus, sketch out two opposite tendencies in the discussion around the human face. The first front conceives the subject as interlocked with the unique expression of his own face. At the same time, this trend towards the expression of the subject through the face is coupled with the counter-tendency of hiding, masking, and dissimulating the expression.

This dichotomy is evident in the research carried out by Jean-Jacques Courtine and Claudine Haroche (1992), who singled out some “paradoxes of the face”: expressing vs. silencing; revealing vs. masking; showing vs. hiding (Courtine, Haroche 1992: 14). I fully endorse the thrust of Courtine and Haroche’s approach that a study of the face should include not only a history of the emergence of the expression of the face but also the silence of the face, so to speak, the ways of controlling, hiding, silencing the human face in different contexts, epochs and cultures (Courtine, Haroche 1992: 20).^10^

From this discussion. it can be gleaned that there are two main modalities of face disguise one static (masking) and one dynamic (mimic and simulation). In the remainder of this study, I will pursue a discussion of the first modality and then conclude by sketching a typology of masks.

## Static dimension of face disguise: mask as displacement of identity

One of the lynchpins of face perception is identity as the face is the visible heart of the individual. Information broadcast through the face is, indeed, multilayered. A whole host of different stimuli are elicited through the face and perceived by others, who constantly monitor faces in others to decode facial stimuli in terms of cues of various nature and having different meanings (Ekman 1978). Identity, gender, age, skin pigmentation, health, basic emotions (Darwin (1965)[1872]), micro-expressions, intentions, and much more information is displayed and expressed – either willingly or unwillingly, inferred and decoded – accurately or less accurately by those who engage in social settings.

Whilst it is visible to others unless masked or disguised, the face is invisible to the subject. Indeed, the face has a twofold character: the face as *seer* and the face as *seen*. Ingold (2002, p. 124) writes,

As a surface, the face has some very peculiar properties. I can feel my own face, and others can see it. But it remains invisible to me. Where others see my face, I see the world. Thus, the face is a visible appearance, in others’ eyes, of my own subjective presence as an agent of perception. It is, if you will, the *look* of a human being.

Thus, the human face is the element of appearance which makes the social bond possible through the responsibility that each individual takes in respect to the outer world, which is the principle of personal identity (Le Breton 2010, p. 72). Given the centrality that the face takes on in social settings (Goffman 1956, 1967; Edkins 2015) and in the definition of one’s own identity (Belting 2017), it is not surprising to find a strong connection between the perception of the human face, the recognition of faces by others, and the pivotal role that such processes play in human interactions and the perception of the environment.

Given the relevance of the element of appearance – showing and being perceived by others – it goes without saying that the opposite tendency – hiding, dissimulating, masking – must be considered in the management of one’s appearance. Indeed, the reverse mechanism of recognition is masking, for it hampers the possibility of recognizing another by altering the distinctive facial traits of the person who wears a mask (Ogibenin 1975). Here we come full circle through the opposite mechanism that lays at the cornerstone of the management of appearance: the simulation and dissimulation of one’s face.

Undoubtedly, the most basic form of altering human appearance, especially the face, is the mask. As said before, the mask makes a face unrecognizable inasmuch as it inhibits the recognition of the individual (Gombrich 1972, p. 9), clouding the informative cues stemming from faces. Masks have been used for millennia for a host of different reasons and embody different functions.

A quick look at the dictionary definitions of the term “mask” suffices to show the variety of its functions, forms, and uses: magical and ritual, war and combat, theatrical and aesthetical, carnivalesque and fun purposes, disguise and deception. In many societies, the use of masks for spiritual purposes seems prevalent (Lommel 1970). Roget’s dictionary defines this term as “a covering for all or part of the face, worn to conceal one’s identity” or “a grotesque or humorous false face worn at a carnival, masquerade, etc.”.^11^

Among the most important functions of masks, we may recall the “protective” and the “intrusive” functions. According to the psychologist Scheibe (1979, p. 67), protection and intrusion refer to the two fields of strategic intelligence: espionage and counter-espionage. For Scheibe (1979, 67), the typical example of a mask as a protective device is the armour. Examples of this type are the face masks used in certain sports, gas masks, oxygen masks and surgical masks, as well as the mask used by the bandit to hide his face.^12^ The mask also has the function of a device designed to penetrate the defences of others in order to gain access to new information (Scheibe 1979, 68), as in the classic example of the Trojan horse.

That there is a nexus between masks and deception is of no doubt, although the functions of masks are not resolved by including this function exclusively. Masks and disguise are related to deceit because such face implements and artifacts establish a fracture and a bipartition, which Lacan refers to as a “fission” of the being (Sini 1993, p. 37). The aspect of duplicating one’s appearance–the schism within the human being–through the mask is important, and it resurfaces in numerous definitions of the mask as a “second face” (Grimes 1975, p. 509), as being “unnatural” (Damisch 1982, p. 787), as a multiplication of faces (Lévi-Strauss 1979), and as “substitution” and “splitting/doubling” (Damish 1982, p. 785). Apropos these various aspects of the mask, both Marin (1993, p. 2) and Hubert Damish (1982, p. 785) are very quick to point out that masks presuppose the characteristics of repeatability and separability, two aspects that parallel the mask to the nature of the sign. Due to these two features, thus, the mask functions as a displacement proxy that allows the wearer of masks to shift between identity and alterity, self and other. Needless to say, the displacement of identity that is proper of the mask yields to a host of different functions that can be fulfilled by wearing a mask.

As a concluding remark in this section, it should be stressed that above all, the face mask is characterized by its nature of being fixed, static and rigid. This point is important and not only fits in with the distinction pointed out above between static and dynamic dimensions in the study of the human face, but it also aligns with the etymological and historical roots of this artifact. Indeed, the use of funeral masks throughout cultures and societies illustrates well this aspect of the mask. The elements of fixity and rigidity of the face mask can be traced to its use in funeral and religious contexts where “dying, like masking, is a rigidifying process,” and death masks embody the experience of “concretion” (Grimes 1975, p. 509).

### Disguise as being in incognito: a typology of masking

The function of displacement of identity, as described above, and its implications and ramifications need some further qualification. Because masking allows a shift between identity and alterity, masks can serve a variety of purposes, one of which is anonymity.

The connection between masking and anonymity is not so obvious because, as pointed out above, the elements of the connotation of masking and disguise are dissembling and deceit. This point is so well laid out in a short but well-document article by Damish (1982, p. 776), where he has argued that being incognito can represent a “zero degree” of the mask. This point is worth pondering. Damish’s point of departure is the historical remark that in Renaissance Venice, the use of “bauta”–the typical Venetian mask made of a black silk hood coupled with a white mask called ‘the face’–was not used for disguise as it was generally employed to achieve a certain degree of anonymity in the life of the city. As he puts it,

The pleasure of the incognito is not to be mistaken for another, but to go around without being recognized and without being identified with anyone other than a mask, or that mask. While disguise is made to deceive, incognito does not impose any identity substitution, but only wants to nullify it (Damish 1982, p. 776).

This is an interesting point because whilst masks as incognito erase identity, masks as disguise substitute identity. Thus, being nobody and pretending to be another are different things and should be distinguished. As Damish well explained, coupled with the degree of anonymity given to the mask wear, goes a certain degree of liberty to people who perform in incognito.

What can we gain from these passages taken together is that Damish perceives the notion of a mask as having at least a double significance that should be spelled out and that feedback into the distinctions laid out in the first part of the present article. By including Damish’s insight on the degree zero of the mask, we could envisage a working typology of disguise that does blur the distinctions laid out above.

**Table 5:** *Typology of masking*

Mask as fiction	Zero degree of the mask	Mask as disguise
Dramatical/Theatrical mask	Anonymity	Dissembling/deceit
Acting/as if	Obliteration of identity	Substitution of identity

## Conclusions

Deception, secrecy, and concealment are very vast, complex, and multidisciplinary subjects of study. The present study investigated and thematized the interrelation between face masking, concealment, and deceit. By drawing on the history of physiognomy, the present study has outlined two main forms of the mask. One is predicated upon the emphasis on the static dimension of the face, and the other is geared upon its dynamic aspect. It was found that classifications of masks elaborated within the field of semiotic theory hitherto, however useful, remain insufficient to encompass the full scope of this phenomenon. For this reason, the study has provided a new typology of masks that includes in the framework the idea of anonymity as a minimal degree of masking.

## Footnotes

1 My translation from French: “Nous remarquerons d’abord qu’un nombre d’*animaux* sont capables de *se cacher*, soit pour échapper à leurs ennemis, soit pour guetter leurs propres proies, et les attaquer par surprise. […] On objectera peut-être qu’il ne s’agit pas d’un mensonge, car cette conduite n’implique évidemment ni paroles, ni référence savante à la notion de vérité. Ma elle joue un rôle primordial dans la lutte pour la vie, que ce soit sous la forme de la défense ou de l’attaque, et elle est peut-être la source et le modèle de tout mensonge, car elle consiste à *modifier la conduite de l’adversaire, en le privant de l’information* dont il a failli disposer : à partir du moment où le prédateur ne perçoit plus sa victime, il devient incapable de la poursuivre. Et tel est, d’une manière tout à fait générale, le processus du mensonge : il s’agit de transformer une situation à son avantage, en modifiant le signes sur lesquels le jugement de l’adversaire pourrait s’exercer” (Durandin 1972, p. 112, italics in original).

2 There is no general agreement about this point. Sommer (1992) holds that deceit is an evolutionary trait that can be found both in human and non-human animals. Other scholars disagree with this view. For instance, Arendt (1972), T. Hobbes and S. Ferenczi argued that “the ability to lie is one of the criteria that distinguish human beings from other animals” (Barnes 1994, p. 3). Likewise, Rappaport (1979, p. 224) holds that “lying is essentially a human problem”. Recent studies have investigated the subject of deception in animal communication with particular focus on the phenomenon of mimicry (Maran 2017). The bibliography on this subject is vast. Useful indications can be found in R. Mitchell and N. Thompson (eds.), *Deception. Perspectives on Human and Nonhuman Deceit*, Albany, State University of New York Press, 1986.

3 The literature on the subject from the standpoint of analytical philosophy, philosophy of language, and linguistics is vast. For a recent account on the subject, see Jennifer M. Saul, *Lying, Misleading, and What is Said. An Exploration in Philosophy of Language and Ethics* (Oxford, Oxford University Press, 2012). See also James E. Mahon “The definition of lying and deception”, in *Standford Encyclopedia of Philosophy*, Standford, Standford University, 2008; A. Isenberg, “Deontology and the ethics of lying”, *Philosophy and Phenomenological Research*, 24/4, 1964, 463–480; Frederick A. Siegler, “Lying”, *American Philosophical Quarterly*, 3, 1966, 128–136; L. Coleman, P. Kay “Prototype semantics: the English word lie”, *Language* 57/1, 1981, 26–44; F. D’Agostini, *Menzogna* (Torino: Bollati Boringhieri, 2012).

4 I am aware that this is perhaps an overgeneralization because, in the history of lying, there were tendencies that counteracted this claim.

5 “If falsehood had, like truth, but one face only, we should be upon better terms; for we should then take as certain the contrary to what the liars say: but the reverse of truth has a hundred thousand forms, and a limitless field, without bond or limit” (Montaigne 1952, p. 16).

6 It is worth noting that some scholars registered a lack of interest in Western scholarship in general about the issue of deception and lying. As D. Sless (1986: 28) states, “It is a curious fact of Western scholarship that there are endless treatises on the subject of truth but few on lying”. This tenet is endorsed by Barnes (1994: 4–5), Bok (2003: 5), Raskin (1987: 445), and Shibles (1985: 24), but it should be recalibrated in light of the recent revival of the current interest in the subject (McGlone and Knapp 2010; Keyes 2004; Callahan 2004).

7 To be accurate, Eco was not the first semiotician who foresaw the potential of lying as a semiotic problem. Likewise, Charles Morris, in discussing the informative function of signs, makes an important remark about the possibility of lying through signs: “Lying is the deliberate use of signs to misinform someone, that is, to produce in someone the belief that certain signs are true which the producer himself believes to be false. The discourse of the liar may be highly convincing. The mere making of false statements is not lying, nor are the forms of misrepresentation lying – as in painting which portrays objects with characteristics that they do not, in fact, have. Lying is connected with the informative function, regardless of which kinds of signs are used for the purpose of misinforming” (Morris 1955 [1946], p. 200).

8 The distinction between non-deceptively intended forms of misrepresentation vs. deceptively intended forms is not a novel distinction and, in fact, was a commonplace in antiquity. This sharp division can be traced back to antiquity under the terminology of *fallax* as opposed to *mendax*.

9 On this point, see also Courtine and Haroche (1992, pp. 156–174).

10 The idea of hiding one’s face and controlling the facial mimic for strategic purposes has a long pedigree, and it finds its best expression in the Renaissance periods.

11 https://www.dictionary.com/browse/mask.

12 Surgical masks have been the object of numerous studies in the last due years due to the massive uses of such devices of protection during the pandemic of Covid 19. See, Leone (2020; 2021), Marini et al. (2021).
